# Genetic parameters for milk urea and its relationship with milk yield and compositions in Holstein dairy cows

**DOI:** 10.1371/journal.pone.0253191

**Published:** 2021-06-18

**Authors:** Hadi Atashi, Miel Hostens, the GplusE consortium

**Affiliations:** 1 Department of Animal Science, Shiraz University, Shiraz, Iran; 2 Department of Farm Animal Health, University of Utrecht, Utrecht, The Netherlands; University of Life Sciences in Lublin, POLAND

## Abstract

The aim was to estimate genetic parameters for milk urea (**MU**) concentration and its relationship with milk yield and compositions in Holstein dairy Cows. Edited data were 90,594 test-day records of milk yield and composition collected during 2015 to 2018 on 13,737 lactations obtained from 7,850 Holstein cows in 50 herds. Random regression test-day model was used to estimate genetic parameters. (Co)variance components were estimated with the Bayesian Gibbs sampling method using a single chain of 400,000 iterates. The first 50,000 iterates of each chain were regarded as a burn-in period. Mean (SD) of MU was 23.03 (5.99) and 22.41 (5.74) mg/dl in primiparous and multiparous cows, respectively. Average heritability estimates for daily MU was 0.33 (SD = 0.02) ranged 0.29 to 0.36 and 0.32 (SD = 0.03) ranged 0.27 to 0.34, respectively, for primiparous and multiparous cows. The mean (SD) genetic correlation between MU and milk yield, fat yield, protein yield, lactose yield, fat percentage, protein percentage, lactose percentage, and somatic cell score was, respectively, -0.02 (0.03), -0.02 (0.01), 0.01 (0.04), 0.01 (0.03), 0.00 (0.07), -0.03 (0.04), 0.00 (0.01), -0.11 (0.06) in primiparous cows. The corresponding values in multiparous cows were -0.01 (0.02), -0.01 (0.03), -0.04 (0.04), -0.04 (0.04), 0.04 (0.04), 0.04 (0.07), -0.03 (0.09), 0.06 (0.11), respectively. The results indicate that selection on MU is possible with no effect on milk yield or compositions, however, relationships between MU and other important traits such as longevity, metabolic diseases, and fertility are needed.

## Introduction

Dairy cows utilize dietary nitrogen for maintenance functions, milk production and tissue growth; however, they do not efficiently convert dietary nitrogen into animal products. Excess nitrogen fed in the form of feed proteins is excreted in urine and feces which is considered as an important environmental concern [1–3]. Milk urea (**MU**) reflects the efficiency of protein synthesis and provides information for dairy producers about the balance between crude protein and energy in the diet [3,4]. The main factor affecting MU is the amount of protein in the diet, however factors including water intake, the time of feeding relative to milking time, stage of lactation, season, genetics, milking frequency, rumen health, and liver function can affect MU [5–7]. MU is a common tool used for evaluation of diet composition and feeding disorders [8,9], and may be related to milk yield and milk compositions as well as reproductive performance, longevity, and health in dairy cows [10–14]. Therefore, the possibility of using MU as a predictor for indirect selection is of great importance [15]. On the other hand, the relationships of MU with nitrogen excretion in milk and urine suggest that decreased MU will decrease environmental pollution with nitrogen [3,8,16,17]. Therefore, milk urea might be used as a selection tool, and information on its genetic parameters is needed. Although genetic parameters for MU have been investigated in a number of studies [2,11,12,18–21], the range in estimates is broad, as are the numbers of animals and the types of models used and gives no clear indication of the heritability or genetic correlations of MU for the dairy cows. Therefore, the aim of this study was to use random regression (RR) test-day models (TDM) to estimate genetic parameters (e.g., heritability and genetic correlation) for MU and its correlation with milk yield and milk compositions in Holstein dairy cows.

## Materials and methods

### Data

Data in this study were collected as part of the Genotype plus Environment (GplusE) FP7-Project (http://www.gpluse.eu). Edited data were 90,594 test-day records of milk yield and milk composition collected during 2015 to 2018 on 13,737 lactations obtained from 7,850 Holstein cows in 50 herds in Belgium and the Netherlands. MU was measured by infrared spectroscopy in milk test-day samples collected and expressed as mg/dl. The age at the first calving (AFC) was calculated as the difference between birth date and calving date at the first parity and was restricted to the range of 540 to 1,200 d. Only records from 5 to 365 days in milk (DIM) were subject to analysis. Test-day somatic cell count (**SCC**) were log transformed to somatic cell score (**SCS**) based on the following equation:

SCS=log2(SCC/100000)+3

Only records from cows that had data for MU and production traits included (milk yield (**MY**), fat yield (**FY**), protein yield (**PY**), lactose yield (**LY**), fat percentage (**FP**), protein percentage (**PP**), lactose percentage (**LP**), and SCS) on a given test day were kept. Within cow, if parity 3 was present, parities 1 and 2 were also present, and if parity 2 was present, parity 1 was also present. Data were divided into two sets of the primiparous (51,345 test-day records on 7,850 cows) and multiparous cows (39,249 test-day records on 4,358 cows). The following random regression (RR) test-day animal model through four-trait three-lactation was used to estimate variance components for test-day records of milk yield and compositions:

$${\text{y}}_{\text{ijklm}}=\mu +{\text{HTD}}_{\text{pi}}+\sum_{\text{b}=0}^{4}{{\text{AS}}_{\text{j}}ø}_{\text{b}}(\text{t})+\sum_{\text{b}=0}^{2}{{\text{a}}_{\text{l}}ø}_{\text{b}}(\text{t})+\sum_{\text{b}=0}^{2}{{\text{pe}}_{\text{l}}ø}_{\text{b}}(\text{t})+{\text{e}}_{\text{ijklm}}$$

where y_ijklm_ is the test-day record (milk yield or milk compositions) on DIM m of cow l in parity k (primiparous and multiparous), belonging to i^th^ class of HTDp, and j^th^ class of AS, HTDp_i_ is the fixed effect of i^th^ class of herd-testday-parity (2,198 classes), AS_k_ is the fixed effect of age-season of calving (112 classes) defined as the following: age at calving in months (ten and seventeen classes of ag at calving were created for primiparous and multiparous cows, respectively) × season of calving (four seasons: winter from Jan-Mar, spring from Apr-Jun, summer from Jul-Sep and autumn from Oct-Dec), $\sum_{\text{b}=0}^{2}{a_{l}ø}_{\text{b}}(\text{t})$ and $\sum_{\text{b}=0}^{2}{{pe}_{l}ø}_{\text{b}}(\text{t})$ are, respectively, the random regression coefficients of additive and permanent environment effects, e_ijklm_ is the residual random effect. Residual random effects were assumed to be normally distributed with the mean of 0.0. The (co)variance components were estimated by Bayesian inference using the Gibbs sampler of the GIBBS3F90 program [22]. Gibbs sampling was used to obtain marginal posterior distributions for the various parameters using a single chain of 400,000 iterates. The first 50,000 iterates of each chain were regarded as a burn-in period to allow sampling from the proper marginal distributions. The length of this burn-in period was determined by visually inspecting plots of sample values across rounds. Homogeneity of residual variance of MU across the lactation was tested by computing the standard deviation (SD) of observed residuals (difference between observed and predicted values) for each DIM in both primiparous and multiparous cows. Genetic (co)variances for each DIM were calculated using the equation described by Jamrozik and Schaeffer [23]. Daily heritability was defined as the ratio of genetic variance to the sum of genetic, permanent environmental, and residual variances at a given DIM. Two-trait multi-lactation random regression model were used to estimate genetic correlation between MU values in primiparous and multiparous cows.

## Results

**Fig 1:**
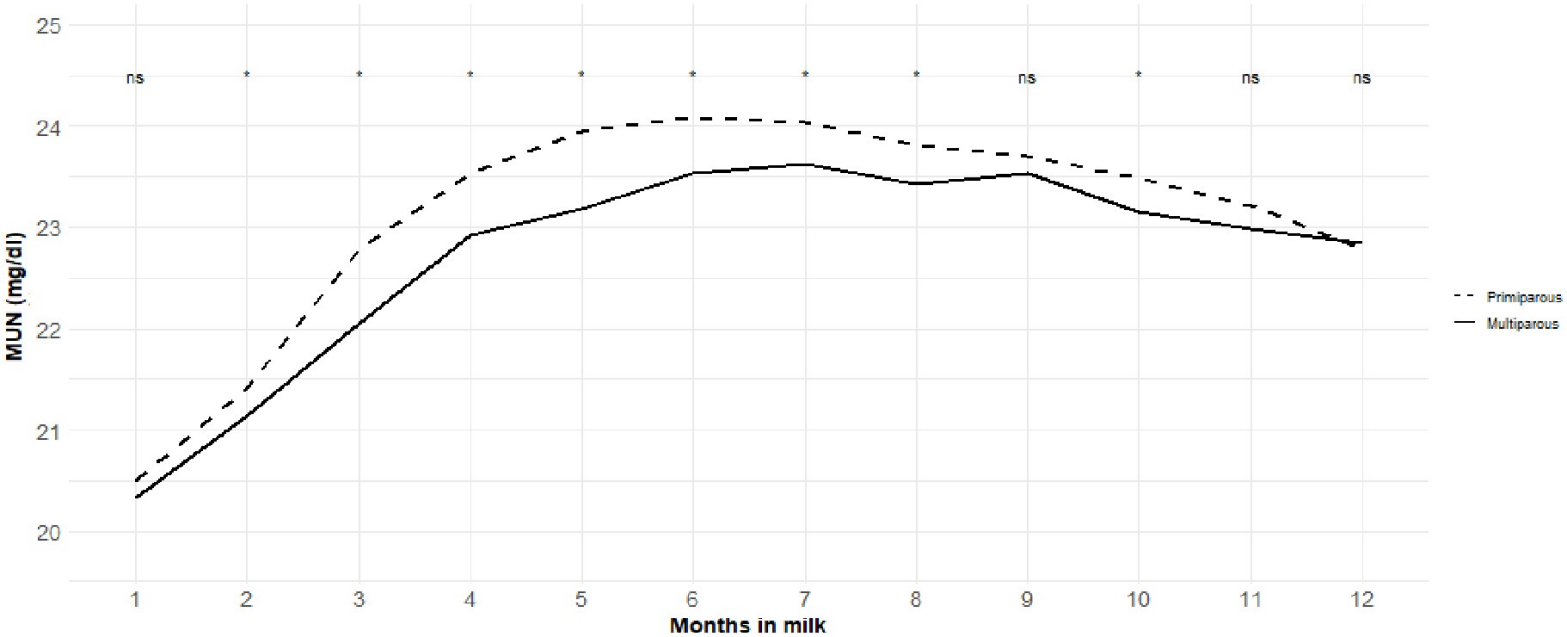
Trends (daily least square means) in concentrations of milk urea across lactation in primiparous and multiparous cows.

**Fig 2:**
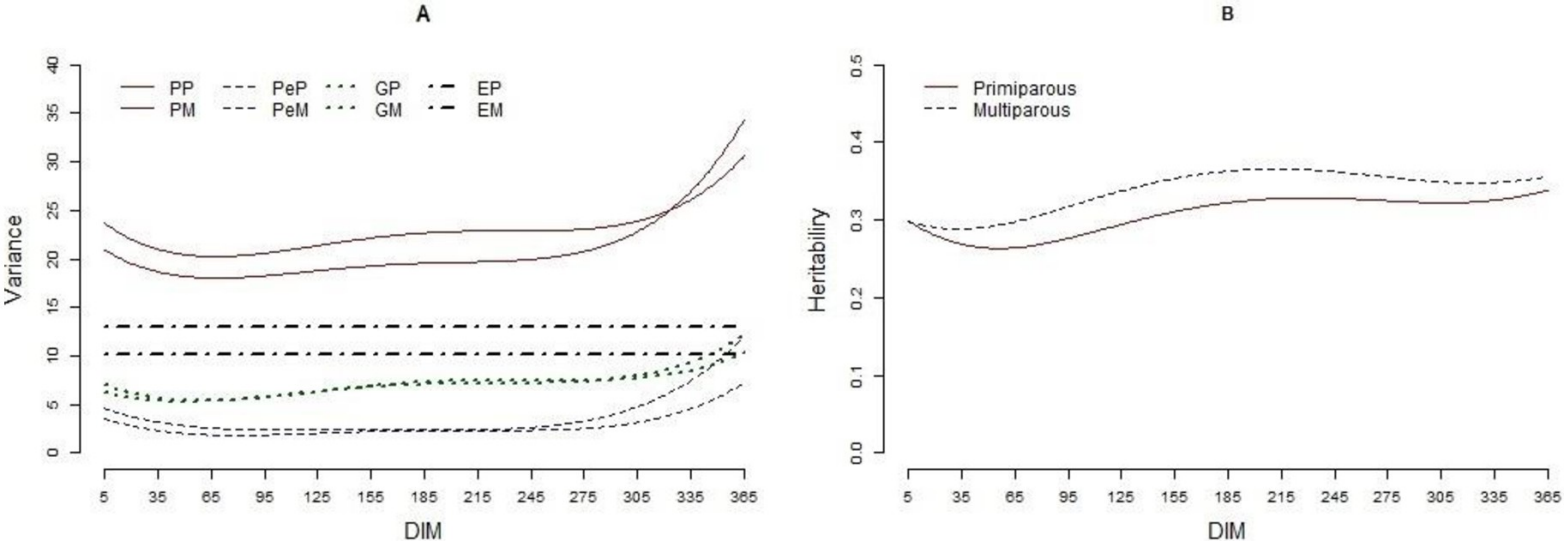
Variance components (A) and heritability (B) of MU in primiparous and multiparous cows. Phenotypic variance in primiparous (PP), phenotypic variance in multiparous (PM), permanent environmental variance in primiparous (PeP), permanent environmental variance in multiparous (PeM), genetic variance in primiparous (GP), genetic variance in multiparous (GM).

**Fig 3:**
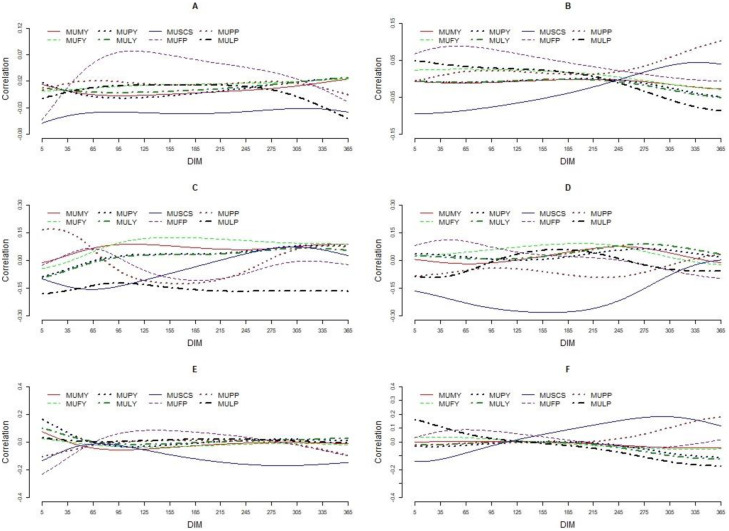
Correlation estimated in daily basis between MU and productive traits included in primiparous and multiparous cows within DIM. Phenotypic correlation in primiparous (A) and multiparous (B), permanent environmental correlation in primiparous (C) and multiparous (D), genetic correlation in primiparous (E) and multiparous (F) cow.

Means, coefficients of variation, minimum, and maximum values for the traits included (MU, MY, FY, PY, LY, FP, PP, LP, and SCS) are presented in Table 1. The mean (SD) MU for primiparous cows of mg/dl 23.03 (5.99) was higher than that for multiparous (22.41 (5.74) mg/dl) cows. Daily MU ranged 1 to 103 and 2 to 84 mg/dl in primiparous and multiparous cows, respectively. The coefficient of variation for MU in the primiparous and multiparous cows was 26%. The lowest value for MU was found at the beginning of lactation, then increased by increasing DIM, reached the peak at the middle of lactation, then decreased by increasing DIM to the end of the lactation (Fig 1). Environmental factors including, herd, calving year, calving month, herd-test-day (HTD), age at the first calving (both in linear and quadratic forms), parity, and months in milk (MIM) affected MU, while in all MIM, except for the MIM 1, 9, 11 and 12, mean MU was higher in primiparous than that in multiparous cows (Fig 1). The variance components and heritability estimated for MU using four-trait multi-lactation are presented in Fig 2. Phenotypic variance for MU was high at the beginning of lactation, decreased by increasing DIM and showed a consistent trend in a major part of the lactation, then increased by increasing DIM at the end of lactation where its highest level was found. Similar trends were found for permanent environmental and genetic variances. Mean additive genetic variance for MU in primiparous and multiparous cow was 7.02 and 7.07 (mg/dl)^2^, respectively. The corresponding values found for permanent environmental variance were 2.71 and 3.63 (mg/dl)^2^, respectively, in primiparous and multiparous cow. Additive genetic variance for MU was high in the beginning of lactation (7.05 and 6.24 (mg/dl)^2^, respectively, in primiparous and multiparous cows), reached the minimum level at DIM 57 (5.34 (mg/dl)^2^) in primiparous and DIM 46 (5.30 (mg/dl)^2^) in multiparous, then increased thereafter until the end of lactation where its maximum level was found (10.34 and 12.16 (mg/dl)^2^, respectively, at DIM 365 in primiparous and multiparous cows). Average heritability estimates for daily MU was 0.33 (SD = 0.02) ranged 0.29 to 0.36 and 0.32 (SD = 0.03) ranged 0.27 to 0.34, respectively, for primiparous and multiparous cows. Means and patterns of phenotypic, permanent environmental, and genetic correlations estimated on a daily basis between MU and production traits included are presented in Table 2 and Fig 3, respectively. Phenotypic correlations between MU and productive traits included were close to zero (ranged -0.04 to 0.04 and -0.03 to 0.04, respectively, in primiparous and multiparous cows). Mean (SD) genetic correlation between MU concentration in primiparous and multiparous cows was 0.83 (0.08) ranged 0.59 to 0.90. The mean (SD) genetic correlation between MU and MU, FY, PY, LY, FP, PP, LP, and SCS was, respectively, -0.02 (0.03), -0.02 (0.01), 0.01 (0.04), 0.01 (0.03), 0.00 (0.07), -0.03 (0.04), 0.00 (0.01), -0.11 (0.06) in primiparous cows. The corresponding values in multiparous cows were -0.01 (0.02), -0.01 (0.03), -0.04 (0.04), -0.04 (0.04), 0.04 (0.04), 0.04 (0.07), -0.03 (0.09), 0.06 (0.11), respectively.

**Table 1:** Mean, minimum, maximum, and CV for MU, and milk production traits in primiparous (n = 51,345 test-day records on 7,850 lactations) and multiparous (n = 39,249 test-day records on 4,358 lactations) cows.

Primiparous					Multiparous			
trait	Mean	Minimum	Maximum	CV (%)	Mean	Minimum	Maximum	CV
MU (mg/dl)	23.03	1.00	103.0	26	22.41	2.00	84.0	26
MY (kg)	27.21	3.00	69.10	23	33.93	3.10	73.50	27
FY (kg)	1.16	0.12	3.15	20	1.44	0.20	3.64	24
PY (kg)	0.97	0.10	2.39	20	1.21	0.15	2.85	23
LY (kg)	1.27	0.12	3.44	24	1.55	0.14	3.44	28
FP (%)	4.35	2.0	6.41	16	4.36	2.1	6.65	17
PP (%)	3.59	2.03	6.93	10	3.63	2.26	6.32	11
LP (%)	4.64	2.59	5.18	4	4.56	2.54	5.17	4
SCS^1^	2.07	0.01	5.30	48	2.20	0.01	5.30	52

^1^. SCC = log2 (SCC/100000) + 3.

**Table 2:** Phenotypic, permanent environmental, and genetic correlations between MU and milk yield and compositions in primiparous and multiparous cows.

	Primiparous			Multiparous		
	P^1^	PE^2^	A^3^	P^1^	PE^2^	A^3^
MY (kg)	0.00	0.07	-0.02	-0.01	0.02	-0.01
FY (kg)	0.02	0.08	-0.02	0.01	0.05	-0.01
PY (kg)	0.01	0.03	0.00	-0.01	0.03	-0.04
LY (kg)	0.00	0.02	0.01	-0.01	0.03	-0.04
FP (%)	0.04	-0.04	0.00	0.04	0.00	0.03
PP (%)	0.01	0.00	-0.02	0.03	-0.06	0.04
LP (%)	0.00	-0.14	0.00	0.00	-0.02	-0.03
SCS^4^	-0.04	-0.04	-0.10	-0.03	-0.19	0.06

^1^. Phenotypic correlation.

^2^. Permanent environmental correlation.

^3^. Additive genetic correlation.

^4^. SCS = log2 (SCC/100000) + 3.

## Discussion

The average MU (23.03 and 22.41 mg/dl in primiparous and multiparous cows, respectively) found in in this study is in close agreement with those reported in the literature [12–15,24] and is within the recommended range of 15.0 to 30.0 mg/dl proposed by [25]. Samoré, Romani [26] reported that mean MU in Italian Brown Swiss dairy cows is 25.9 mg/dL. Lopez-Villalobos, Correa-Luna [19] reported that mean MU in in New Zealand grazing dairy cows is 24.9 (mg/dl). The mean MU for primiparous cows was higher than that for multiparous, even though the difference was small, which is in an agreement with previous studies [18,20,27]. Higher MU concentration in primiparous cows may be associated with lower body weight and lower level of milk yield in primiparous cows when compared to multiparous cow [8]. Different lactation curve patterns including a curve resembled the lactation curve for milk [8,24] or a mirror shape [15,21] has been reported for MU. In the current study, the lowest value for MU was found at the beginning of lactation, increased with increasing DIM, reached the peak in the middle part of the lactation, then decreased with increasing DIM to the end of the lactation. Within the study of Mucha and Strandberg [12] MU was lowest at the beginning of lactation and reached the peak at around 75 DIM, where it remained until 180 DIM and was only slightly decreasing until the end of lactation. Rzewuska and Strabel [20] reported that MU was the lowest during the first month of lactation and its peak occurred at the fifth month of lactation in Polish Holstein dairy cows. In contrast, Wood, Boettcher [15] reported that MU levels decreased to a minimum level at the second months in milk (**MIM**), then increased until the end of lactation. The difference in lactation curve found for MU may be due to management or nutrition, although no clear explanation can be given [12]. The coefficient of variation for MU in primiparous and multiparous cows was 26%. Samoré, Romani [26] reported that the coefficients of variation for MU in Italian Brown Swiss dairy cows was 27%. Rzewuska and Strabel [20] reported that the coefficient of variation for MU ranged 40 to 41% in Polish Holstein cows. Lopez-Villalobos, Correa-Luna [19] reported that the coefficient of variation for MU in grazing dairy cattle in New Zealand is 36.7%. Environmental factors including, herd, calving year, calving month, herd-test-day (HTD), age at the first calving (both in linear and quadratic forms), parity, and months in milk (MIM) affected MU which is in line with previous studies [15,28,29]. Wood, Boettcher [15] reported HTD and stage of lactation as important factors affecting MU level in Holstein cows. The association between MU and cow age, season and parity was also documented in previous studies [15,28,30]. Phenotypic, permanent environmental, and genetic variances for MU were higher at the beginning and the end of lactation. The higher variance observed in the beginning of lactation can be attributed to the large variations in milk compositions, the rapidly changing ration, and the adaptation of the rumen to a high-production ration in this period of lactation [31,32]. Mean heritability estimates for daily MU for primiparous and multiparous cows was 0.33 and 0.32, respectively. Miglior, Sewalem [21] reported that the heritability for MU ranged 0.38 to 0.41. Wood, Boettcher [15] reported that heritability estimates for MU in the first three parities ranged between 0.44 to 0.59. Mucha and Strandberg [12] reported that heritability for MU was between 0.16 and 0.18 across lactation. Samoré, Romani [26] reported that MU had a heritability of 0.17 in Italian Brown Swiss dairy cattle. Beatson, Meier [2] reported that mean heritability estimates for MU is 0.28. Rzewuska and Strabel [20] reported that mean heritability for MU in Polish Holstein cows is 0.22. The variation found for heritability of MU in the literature can be explained, at lease in a part, by the differences in structure of the data, such as herd size, number of records per cow, number of cows per sire, and length of the period of data collection. MU had a low phenotypic correlation with milk yield and milk compositions in a close agreement with Miglior, Sewalem [21]. The current research used a random regression model, which allowed changes of correlation during lactation. The results showed that genetic correlations between MU with MY and milk compositions (FY, PY, LY, FP, PP, LP) were close to zero which is in a close agreement with Wood, Boettcher [15]. Genetic correlation between MU and productive traits has been investigated by a number of researchers, however the reported results are inconsistent. Godden, Lissemore [28] reported a positive genetic correlation between MY and MU, whereas others reported a negative relationship [30,33]. A number of researchers reported negative genetic correlation between MU and PY [28,30], while there are many researchers who reported a positive genetic correlation between MU and PY [24]. Mucha and Strandberg [12] reported that genetic correlation of MU with MY, FY, and PY were weakly positive until 230 DIM and negative thereafter, ranging from 0.22 to −0.15 from the beginning to the end of lactation. Samoré, Romani [26] reported that MU had a positive genetic relationship with FY (0.12), null with PY (0.03) and a negative genetic correlation with MY (-0.17) in Italian Brown Swiss dairy cattle. Genetic correlation between MU and SCS ranged -0.17 to 0.01 and -0.14 to 0.18, respectively, in primiparous and multiparous cows. Mean genetic correlation between MU and SCS was -0.11 in primiparous cows which is in line with Godden, Lissemore [28] who reported a negative genetic correlation between MU and SCC. Miglior, Sewalem [21] reported that genetic correlation between MU abs SCS is −0.19. However, mean genetic correlation between MU and milk SCS was 0.06 in multiparous cows. Samoré, Romani [26] reported that MU was not genetically correlated with milk SCC in Italian Brown Swiss dairy cattle. Lopez-Villalobos, Correa-Luna [19] using a small number of test-day records (n = 1,284) reported that mean genetic correlation between MU and MY, FY, PY, LY, and SCS was, respectively, 0.38, -0.21, 0.01, 0.30 and 0.20. Rzewuska and Strabel [20] reported that genetic correlations between MU and MY, FY, PY and SCS ranged, respectively, from 0.20 to 0.42, 0.16 to 0.35, 0.09 to 0.33, and -0.14 to -0.09 in Polish Holstein cows. The variation found for genetic correlation of MU with productive traits in the literature can be explained by the differences in structure of the data, number of records, the statistical models used, and the length of the period of data collection. Mean genetic correlation between MU values in primiparous and multiparous cows was 0.83. Miglior, Sewalem [21] reported genetic correlation of MU among parities is 0.82.

## Conclusion

Milk urea (MU) can be considered as an indicator to monitor the nutritional status of dairy cows and reduce nitrogen emissions to the environment and is included as a standard part in most milk recording systems. This study showed a moderate heritability for MU and a close to zero genetic correlations between MU and production traits. Based on the finding of this study it can be concluded that selection on MU is possible with no effect on milk yield or milk compositions. compositions. The findings of this study can be used as the first step for the development of a routine genetic evaluation for MU and its inclusion into the genetic selection program in Holstein dairy cows, however relationships between MU and other economically important traits such as longevity, metabolic diseases and fertility are needed.

## Supporting information

S1 Dataset Test-day records of milk yield and composition collected during 2015 to 2018 on 13,737 lactations obtained from 7,850 cows in 50 herds were used to estimated genetic parameters for milk urea and its relationship with milk yield and compositions in Holstein cows. (TXT)
